# 4,5-Di­amino-3-[(*E*,*E*)-4-(4,5-di­amino-4*H*-1,2,4-triazol-3-yl)buta-1,3-dien­yl]-4*H*-1,2,4-triazol-1-ium chloride

**DOI:** 10.1107/S1600536813016589

**Published:** 2013-06-22

**Authors:** Roberto Centore, Vincenzo Piccialli

**Affiliations:** a Dipartimento di Scienze Chimiche, Università degli Studi di Napoli ’Federico II’, Complesso di Monte S. Angelo, Via Cinthia, 80126 Napoli, Italy

## Abstract

The title compound, C_8_H_13_N_10_^+^·Cl^−^, is the monochlorhydrate salt of an aromatic bis­(di­amino­triazole). The cation is centrosymmetric, lying about an inversion centre (*C_i_* symmetry) because the acidic H atom is disordered over two centrosymmetrically related ring N atoms, with equal multiplicity. It is noteworthy that protonation occurs at an N atom of the ring, instead of at the C—NH_2_ or N—NH_2_ amino groups. The chloride anions are also in special positions, as they lie on binary axes, and so the crystallographically independent unit contains half of a formula unit. The N atom of the C—NH_2_ group is *sp* ^2^-hybridized and the amino group is coplanar with the triazole ring [dihedral angle = 5 (3)°], while the N atom of the N—NH_2_ amino group is pyramidal. The C=C bonds are in *E* conformations and the cation is flat because the conformation of the carbon chain is fully extended. The chloride anions are hexa­coordinated, in a distorted trigonal–prismatic geometry, and they are involved, as acceptors, in six hydrogen bonds. Chains of hydrogen-bonded cations, running along *c* and *a* + *c*, are generated by *c*-glide and *C*_2_ rotation, respectively. This combination of N—H⋯Cl and N—H⋯N hydrogen bonds leads to the formation of a three-dimensional network.

## Related literature

For semiconductor, optoelectronic and piezoelectric materials containing heterocycles, see: Wen & Liu (2010 ▶); Centore, Ricciotti *et al.* (2012 ▶); Centore, Concilio *et al.* (2012 ▶). For the structural analysis of conjugation in organic mol­ecules containing N-rich heterocycles, see: Carella, Centore, Fort *et al.* (2004 ▶); Centore, Fusco, Capobianco *et al.* (2013 ▶). For the synthesis of related compounds, see: Centore *et al.* (2011 ▶). For the local packing modes of heterocycles containing nitro­gen, see: Centore *et al.* (2013*a* ▶,*b* ▶). For H bonding in crystal structures, see: Centore, Fusco, Jazbinsek *et al.* (2013 ▶). For the crystal structure of the dichlorhydrate salt, see: Centore, Fusco, Carella & Causà (2013 ▶). 
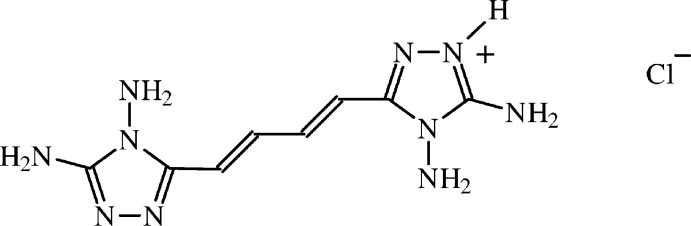


## Experimental

### 

#### Crystal data

C_8_H_13_N_10_^+^·Cl^−^
*M* *_r_* = 284.73
Monoclinic, 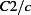

*a* = 10.360 (3) Å
*b* = 10.823 (4) Å
*c* = 11.123 (4) Å
β = 98.27 (2)°
*V* = 1234.2 (7) Å^3^
*Z* = 4
Mo *K*α radiation
μ = 0.32 mm^−1^
*T* = 293 K
0.40 × 0.10 × 0.08 mm

#### Data collection

Bruker–Nonius KappaCCD diffractometer
Absorption correction: multi-scan (*SADABS*; Bruker, 2001 ▶) *T*_min_ = 0.884, *T*_max_ = 0.977
4820 measured reflections
1405 independent reflections
999 reflections with *I* > 2σ(*I*)
*R*_int_ = 0.048

#### Refinement

*R*[*F* ^2^ > 2σ(*F* ^2^)] = 0.047
*wR*(*F* ^2^) = 0.128
*S* = 1.05
1405 reflections
102 parameters
H atoms treated by a mixture of independent and constrained refinement
Δρ_max_ = 0.28 e Å^−3^
Δρ_min_ = −0.37 e Å^−3^

### 

Data collection: *COLLECT* (Nonius, 1999 ▶); cell refinement: *DIRAX/LSQ* (Duisenberg *et al.*, 2000 ▶); data reduction: *EVALCCD* (Duisenberg *et al.*, 2003 ▶); program(s) used to solve structure: *SIR97* (Altomare *et al.*, 1999 ▶); program(s) used to refine structure: *SHELXL97* (Sheldrick, 2008 ▶); molecular graphics: *ORTEP-3 for Windows* (Farrugia, 2012 ▶) and *Mercury* (Macrae *et al.*, 2006 ▶); software used to prepare material for publication: *WinGX* (Farrugia, 2012 ▶).

## Supplementary Material

Crystal structure: contains datablock(s) global, I. DOI: 10.1107/S1600536813016589/bx2445sup1.cif

Structure factors: contains datablock(s) I. DOI: 10.1107/S1600536813016589/bx2445Isup2.hkl

Supplementary material file. DOI: 10.1107/S1600536813016589/bx2445Isup3.cml

Additional supplementary materials:  crystallographic information; 3D view; checkCIF report

## Figures and Tables

**Table 1:** Hydrogen-bond geometry (Å, °)

*D*—H⋯*A*	*D*—H	H⋯*A*	*D*⋯*A*	*D*—H⋯*A*
N2—H2⋯N2^i^	0.80 (5)	1.97 (5)	2.695 (4)	151 (5)
N4—H4*B*⋯N1^ii^	0.90 (3)	2.29 (3)	3.100 (3)	149 (2)
N4—H4*A*⋯Cl1^iii^	0.84 (3)	2.83 (3)	3.652 (3)	165 (3)
N5—H5*A*⋯Cl1^iv^	0.87 (3)	2.79 (3)	3.534 (2)	144 (2)
N5—H5*B*⋯Cl1	0.95 (3)	2.37 (3)	3.265 (2)	156 (2)

Symmetry codes: (i) 
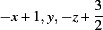
; (ii) 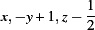; (iii) 
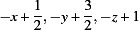
; (iv) 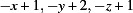.

**Fig. 1.**
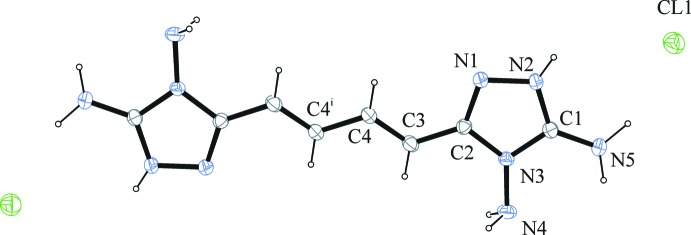
ORTEP view of the molecular structure of the title compound. Thermal ellipsoids are drawn at 30% probability level. The acidic H atom is disordered over N2 and N2i (i= -x + 1/2, -y + 1/2, -z + 1) with occupancy factor 0.5. The chloride anion also has occupancy factor 1/2, as it lies on C2 axes.

**Fig. 2.**
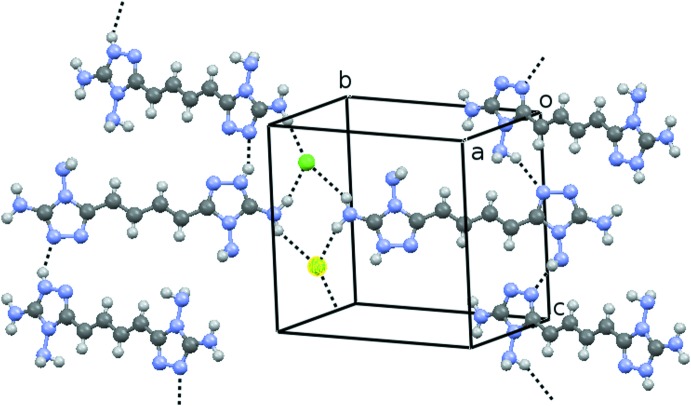
Partial crystal packing of the title compound, showing some H bonding patterns. H bonds are represented by dashed lines. Only one position of the disordered N+–H hydrogen atom is shown.

## supplementary crystallographic information

### Comment

Nitrogen rich, aromatic heterocycles have interest in the field of organic electronics for several reasons. One reason is that the Bondi van der Waals radius of nitrogen is 1.55 Å, as compared with 1.70 Å of carbon, so while the limiting interlayer distance for an all-carbon-containing planar structure is that of graphite (3.4 Å), in planar structures containing *sp^2^* hybridized nitrogen atoms, interplanar distances of 3.1 Å can be reached in principle. Another feature of *sp^2^* nitrogen atoms in heteroaromatic compounds is their capability of acting as H bonding acceptors, a feature that, if properly exploited, can lead to tighter packings. Following our interest in the synthesis of aromatic heterocycles for advanced applications in electronics, optoelectronics and photonics (Carella, Centore, Fort *et al.*, 2004; Centore, Ricciotti *et al.*, 2012; Centore, Concilio *et al.*, 2012), we have recently developed the synthesis on N-rich heterocycles containing the 3,4-diamino-1,2,4-triazole unit (Centore *et al.*, 2011; Centore, Fusco, Capobianco *et al.*, 2013). In the present paper we report the structural investigation of the title compound, shown in the Scheme, which is the monochlorhydrate salt of a bis(3,4-diamino-1,2,4- triazole).

The molecular structure is shown in Fig. 1. The cation is formed by protonation at N2 atom of the ring, instead of C–NH_2_ or N—NH_2_ amino groups. The acidic H atom is found bonded both to N2 and to N2^i^ (i= -*x* + 1/2, -*y* + 1/2, -*z* + 1) with equal multiplicity which is 0.5 since the cation lies in special position on crystallographic inversion centres and has (statistically) *C_i_* symmetry. The chloride anion also lies in special position on *C_2_* axes and has fixed occupancy factor 0.5 too. The geometry around N5 atom is substantially planar indicating *sp*^2^ hybridization (the sum of valence angles at N5 is 360 (3)°) and the plane of the amino group is coplanar with the triazole ring. This suggests partial delocalization of the lone pair of N5 towards the triazole ring, as confirmed by the short length of the bond C1–N5 (1.329 (3) Å). The geometry around N4, on the other hand, is pyramidal (the sum of valence angles at N4 is 318 (4)°). With exception for the two H atoms bonded to N4, the molecule is substantially flat, as the result of the torsion angles of the hydrocarbon chain joining the two rings. The double bonds in the hydrocarbon chain are in *E* configuration.

The molecules of the title compound have several H bonding donor and acceptor groups, and the crystal packing is dominated by the formation of H bonds, Table 1. Several H bonding motifs are recognized in the crystal packing and some of them are shown in Figs. 2. The chloride anion is hexacoordinated, according to a distorted trigonal prismatic geometry; it is involved, as acceptor, in six H bonds: four are formed with C–NH_2_ donors and two with N–NH_2_ donors. Ring patterns *R*^2^_3_(10) and *R*^2^_4_(8) are observed.

Cations are involved in the formation of different H bonded chains. Chains running along *c* are generated by the *c*-glide reflection through H bonding between a N–NH_2_ donor and N1 acceptor of another glide-related molecule. The graph-set symbol of the H bonding pattern is *C*(5). Chains running along *a* + *c* are formed by H bonding between N2–H donor and N2 acceptor of a *C*_2_ related molecule. The graph-set symbol of the H bonding pattern is *C*(11). It is a remarkable finding that the crystal structure of the dichlorhydrate salt is completely different, showing the formation of π-stacked infinite planar layers (Centore, Fusco, Carella & Causà, 2013).

### Experimental

The title compound was prepared by suspending *E,E*-1,4-bis(3,4-diamino-1,2,4-triazol-5-yl)-1,3-butadiene (Centore *et al.*, 2011) in diluted hydrochloric acid (0.1 *M*). By heating the suspension at ebullition, a clear solution was obtained. By adding ethanol, single crystals of the chlorhydrate were obtained by slow cooling to room temperature.

### Refinement

The H atoms bonded to N atoms were located in difmaps and their coordinates were refined. All other H atoms were generated stereochemically and were refined by the riding model. For all H atoms *U*_iso_=1.2×*U*_eq_ of the carrier atom was assumed. The cation (C_8_H_13_N_10_)^+^ is not centrosymmetric by itself. However, since the acidic H atom is equally shared by N2 and N2^i^ (i= -*x* + 1/2, -*y* + 1/2, -*z* + 1) the cation is statistically centrosymmetric (*C_i_* crystallographic symmetry). For this reason the occupancy factor of the H atom bonded to N2 was fixed at 0.5. Chloride anions lie in special positions on binary axes and they too were given fixed occupancy factor 0.5.

### Crystal data

**Table:**

C_8_H_13_N_10_^+^·Cl^−^	*F*(000) = 592
*M**_r_* = 284.73	*D*_x_ = 1.532 Mg m^−^^3^
Monoclinic, *C*2/*c*	Mo *K*α radiation, λ = 0.71073 Å
Hall symbol: -C 2yc	Cell parameters from 150 reflections
*a* = 10.360 (3) Å	θ = 3.7–19.8°
*b* = 10.823 (4) Å	µ = 0.32 mm^−^^1^
*c* = 11.123 (4) Å	*T* = 293 K
β = 98.27 (2)°	Prism, pale brown
*V* = 1234.2 (7) Å^3^	0.40 × 0.10 × 0.08 mm
*Z* = 4	

### Data collection

**Table:**

Bruker–Nonius KappaCCD diffractometer	1405 independent reflections
Radiation source: normal-focus sealed tube	999 reflections with *I* > 2σ(*I*)
Graphite monochromator	*R*_int_ = 0.048
Detector resolution: 9 pixels mm^-1^	θ_max_ = 27.5°, θ_min_ = 3.1°
CCD rotation images, thick slices scans	*h* = −12→13
Absorption correction: multi-scan (*SADABS*; Bruker, 2001)	*k* = −14→12
*T*_min_ = 0.884, *T*_max_ = 0.977	*l* = −14→14
4820 measured reflections	

### Refinement

**Table:**

Refinement on *F*^2^	Primary atom site location: structure-invariant direct methods
Least-squares matrix: full	Secondary atom site location: difference Fourier map
*R*[*F*^2^ > 2σ(*F*^2^)] = 0.047	Hydrogen site location: inferred from neighbouring sites
*wR*(*F*^2^) = 0.128	H atoms treated by a mixture of independent and constrained refinement
*S* = 1.05	*w* = 1/[σ^2^(*F*_o_^2^) + (0.057*P*)^2^ + 1.1655*P*] where *P* = (*F*_o_^2^ + 2*F*_c_^2^)/3
1405 reflections	(Δ/σ)_max_ < 0.001
102 parameters	Δρ_max_ = 0.28 e Å^−^^3^
0 restraints	Δρ_min_ = −0.37 e Å^−^^3^

### Special details

**Table:**

Geometry. All e.s.d.'s (except the e.s.d. in the dihedral angle between two l.s. planes) are estimated using the full covariance matrix. The cell e.s.d.'s are taken into account individually in the estimation of e.s.d.'s in distances, angles and torsion angles; correlations between e.s.d.'s in cell parameters are only used when they are defined by crystal symmetry. An approximate (isotropic) treatment of cell e.s.d.'s is used for estimating e.s.d.'s involving l.s. planes.
Refinement. Refinement of *F*^2^ against ALL reflections. The weighted *R*-factor *wR* and goodness of fit *S* are based on *F*^2^, conventional *R*-factors *R* are based on *F*, with *F* set to zero for negative *F*^2^. The threshold expression of *F*^2^ > σ(*F*^2^) is used only for calculating *R*-factors(gt) *etc*. and is not relevant to the choice of reflections for refinement. *R*-factors based on *F*^2^ are statistically about twice as large as those based on *F*, and *R*- factors based on ALL data will be even larger.

### Fractional atomic coordinates and isotropic or equivalent isotropic displacement parameters (Å^2^)

**Table:**

	*x*	*y*	*z*	*U*_iso_*/*U*_eq_	Occ. (<1)
C1	0.41283 (19)	0.7142 (2)	0.5276 (2)	0.0278 (5)	
C2	0.3433 (2)	0.5222 (2)	0.51757 (19)	0.0276 (5)	
C3	0.2841 (2)	0.4103 (2)	0.4657 (2)	0.0323 (5)	
H3	0.2454	0.4122	0.3850	0.039*	
C4	0.2814 (2)	0.3050 (2)	0.5256 (2)	0.0307 (5)	
H4	0.3225	0.3021	0.6056	0.037*	
N1	0.38783 (18)	0.54308 (17)	0.63082 (16)	0.0313 (5)	
N2	0.43126 (19)	0.66519 (17)	0.63755 (18)	0.0317 (5)	
H2	0.473 (5)	0.691 (5)	0.699 (4)	0.038*	0.50
N3	0.35804 (16)	0.62667 (16)	0.45004 (16)	0.0267 (4)	
N4	0.3270 (2)	0.6457 (2)	0.32470 (18)	0.0372 (5)	
H4A	0.248 (3)	0.630 (3)	0.304 (3)	0.045*	
H4B	0.370 (3)	0.585 (3)	0.292 (3)	0.045*	
N5	0.4393 (2)	0.82900 (19)	0.4965 (2)	0.0365 (5)	
H5A	0.428 (3)	0.849 (3)	0.420 (3)	0.044*	
H5B	0.477 (3)	0.884 (3)	0.558 (3)	0.044*	
Cl1	0.5000	0.98424 (10)	0.7500	0.0552 (3)	

### Atomic displacement parameters (Å^2^)

**Table:**

	*U*^11^	*U*^22^	*U*^33^	*U*^12^	*U*^13^	*U*^23^
C1	0.0261 (10)	0.0267 (12)	0.0304 (11)	0.0013 (8)	0.0038 (8)	−0.0021 (9)
C2	0.0283 (10)	0.0270 (12)	0.0273 (11)	0.0004 (8)	0.0031 (8)	0.0020 (9)
C3	0.0352 (11)	0.0319 (13)	0.0282 (11)	−0.0011 (9)	−0.0003 (9)	−0.0045 (10)
C4	0.0351 (11)	0.0286 (12)	0.0279 (11)	−0.0017 (9)	0.0027 (8)	−0.0044 (9)
N1	0.0376 (10)	0.0278 (10)	0.0274 (10)	−0.0046 (8)	0.0008 (8)	0.0008 (8)
N2	0.0393 (10)	0.0249 (10)	0.0291 (10)	−0.0053 (8)	−0.0009 (8)	−0.0011 (8)
N3	0.0312 (9)	0.0259 (10)	0.0225 (9)	0.0021 (7)	0.0019 (7)	0.0002 (7)
N4	0.0486 (12)	0.0399 (12)	0.0226 (10)	0.0037 (10)	0.0031 (8)	0.0003 (9)
N5	0.0492 (12)	0.0249 (11)	0.0350 (11)	−0.0049 (8)	0.0046 (9)	0.0020 (9)
Cl1	0.0648 (6)	0.0474 (6)	0.0497 (6)	0.000	−0.0042 (5)	0.000

### Geometric parameters (Å, º)

**Table:**

C1—N2	1.321 (3)	C4—H4	0.9300
C1—N5	1.329 (3)	N1—N2	1.395 (3)
C1—N3	1.350 (3)	N2—H2	0.80 (5)
C2—N1	1.298 (3)	N3—N4	1.400 (3)
C2—N3	1.378 (3)	N4—H4A	0.84 (3)
C2—C3	1.440 (3)	N4—H4B	0.90 (3)
C3—C4	1.322 (3)	N5—H5A	0.87 (3)
C3—H3	0.9300	N5—H5B	0.95 (3)
C4—C4^i^	1.434 (4)		
			
N2—C1—N5	127.5 (2)	C1—N2—N1	109.15 (18)
N2—C1—N3	107.5 (2)	C1—N2—H2	129 (4)
N5—C1—N3	124.9 (2)	N1—N2—H2	121 (4)
N1—C2—N3	109.54 (19)	C1—N3—C2	107.25 (18)
N1—C2—C3	127.4 (2)	C1—N3—N4	123.27 (19)
N3—C2—C3	123.1 (2)	C2—N3—N4	129.47 (19)
C4—C3—C2	124.2 (2)	N3—N4—H4A	109 (2)
C4—C3—H3	117.9	N3—N4—H4B	104.1 (18)
C2—C3—H3	117.9	H4A—N4—H4B	105 (3)
C3—C4—C4^i^	123.8 (3)	C1—N5—H5A	118.8 (19)
C3—C4—H4	118.1	C1—N5—H5B	118.1 (17)
C4^i^—C4—H4	118.1	H5A—N5—H5B	123 (3)
C2—N1—N2	106.51 (18)		
			
N1—C2—C3—C4	−9.0 (4)	N2—C1—N3—C2	0.3 (2)
N3—C2—C3—C4	172.4 (2)	N5—C1—N3—C2	−178.3 (2)
C2—C3—C4—C4^i^	177.9 (3)	N2—C1—N3—N4	−178.77 (19)
N3—C2—N1—N2	0.8 (2)	N5—C1—N3—N4	2.7 (3)
C3—C2—N1—N2	−177.9 (2)	N1—C2—N3—C1	−0.7 (2)
N5—C1—N2—N1	178.7 (2)	C3—C2—N3—C1	178.1 (2)
N3—C1—N2—N1	0.2 (2)	N1—C2—N3—N4	178.3 (2)
C2—N1—N2—C1	−0.6 (2)	C3—C2—N3—N4	−3.0 (3)

Symmetry code: (i) −*x*+1/2, −*y*+1/2, −*z*+1.

### Hydrogen-bond geometry (Å, º)

**Table:**

*D*—H···*A*	*D*—H	H···*A*	*D*···*A*	*D*—H···*A*
N2—H2···N2^ii^	0.80 (5)	1.97 (5)	2.695 (4)	151 (5)
N4—H4*B*···N1^iii^	0.90 (3)	2.29 (3)	3.100 (3)	149 (2)
N4—H4*A*···Cl1^iv^	0.84 (3)	2.83 (3)	3.652 (3)	165 (3)
N5—H5*A*···Cl1^v^	0.87 (3)	2.79 (3)	3.534 (2)	144 (2)
N5—H5*B*···Cl1	0.95 (3)	2.37 (3)	3.265 (2)	156 (2)

Symmetry codes: (ii) −*x*+1, *y*, −*z*+3/2; (iii) *x*, −*y*+1, *z*−1/2; (iv) −*x*+1/2, −*y*+3/2, −*z*+1; (v) −*x*+1, −*y*+2, −*z*+1.
